# Modifiable lifestyle factors and sudden cardiac death: mechanisms, evidence, and prevention strategies

**DOI:** 10.3389/fcvm.2026.1870365

**Published:** 2026-07-29

**Authors:** Jinhua Zhang, Xiaowei Meng, Hyungsoo Shin, Wansuk Choi

**Affiliations:** 1 Laiwu Vocational and Technical College, Jinan, Shandong, China; 2 Graduate School of Physical Therapy, Kyungwoon University, Gumi, Gyeongsangbuk-do, Republic of Korea; 3 Department of Cardiology, The Fourth People’s Hospital of Jinan, Jinan, Shandong, China

**Keywords:** autonomic dysfunction, cardiovascular prevention, modifiable lifestyle factors, obstructive sleep apnea, physical activity, sudden cardiac death, ventricular arrhythmia, wearable devices

## Abstract

Sudden cardiac death (SCD) accounts for an estimated 4–5 million deaths annually worldwide and remains a major unresolved challenge in cardiovascular medicine. Survival from out-of-hospital cardiac arrest remains below 10% in most regions, and approximately half of SCD events occur as the first clinical manifestation of cardiovascular disease, often in individuals not identified by conventional risk stratification. We conducted a narrative review of the literature in PubMed/MEDLINE, Web of Science, and EMBASE (January 2003 to April 2026), prioritizing prospective cohorts, randomized trials, meta-analyses, and international guidelines. Five major modifiable lifestyle domains are consistently linked to SCD risk: sleep health, physical activity, diet, psychological stress, and tobacco and alcohol use. Sleep duration shows a U-shaped association with cardiovascular mortality, and untreated obstructive sleep apnea may confer substantially elevated risk (subgroup OR 3.87; 95% CI 1.09–13.81), although overall evidence remains heterogeneous. Physical activity also follows a U-shaped pattern, with habitual moderate activity being protective, and brief vigorous incidental activity (∼4 min/day) reducing cardiovascular mortality by 32%–34%, while sedentary behavior and unaccustomed vigorous exertion increase risk. Mediterranean dietary patterns reduce major cardiovascular events by approximately 30%, and psychological stress promotes arrhythmogenesis through neurohormonal activation and adverse remodeling. These exposures converge on four key mechanisms: autonomic dysfunction, systemic inflammation, structural and electrophysiological remodeling, and metabolic-hormonal dysregulation. Integrating lifestyle-based interventions into routine cardiovascular risk assessment offers a low-cost and scalable strategy for primary prevention. Emerging digital health technologies, including AI-enhanced electrocardiography and consumer-grade wearables, provide complementary opportunities for individualized risk prediction and population-level monitoring, particularly in resource-limited settings where conventional device-based prevention remains limited. Given the observational nature of most included evidence and the heterogeneity of SCD definitions across studies, the mechanistic and clinical inferences presented here should be regarded as hypothesis-generating, motivating prospective and where feasible interventional research rather than immediate practice change.

## GRAPHICAL ABSTRACT

**Figure:**
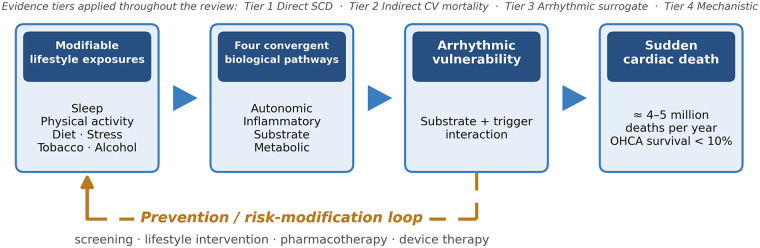
A simplified linear four-node causal chain summarising the pathway from modifiable lifestyle exposures → convergent biological mechanisms → arrhythmic vulnerability → sudden cardiac death, together with a parallel prevention loop labelled ‘Prevention/risk-modification loop'.

## 1 Introduction

Sudden cardiac death (SCD) was historically defined by the World Health Organization (WHO) as an unexpected natural death from a cardiac cause occurring within one hour of symptom onset in a person with or without pre-existing heart disease (1). It represents one of the most catastrophic manifestations of cardiovascular disease. The 2023 Lancet Commission on SCD estimated that 4–5 million individuals die from SCD annually worldwide — a burden exceeding that of any individual cancer and second only to all cancers combined (2). In China, a four-region surveillance study estimated an annual SCD burden of approximately 500,000 cases, based on a crude incidence rate of 41.8 per 100,000 person-years, with updated extrapolations suggesting 540,000–550,000 cases annually, representing the highest absolute burden globally (3). Subsequent epidemiological refinement has broadened the WHO definition to include unwitnessed deaths in individuals last seen alive within 24 h, a definition adopted in the 2022 ESC Guidelines for ventricular arrhythmias and SCD prevention (4).

Despite this substantial burden, the effectiveness of current prevention strategies remains limited. Survival from out-of-hospital cardiac arrest (OHCA) remains below 10% in most regions (2). Contemporary prevention strategies, centered on pharmacological therapy and implantable cardioverter-defibrillators (ICDs) guided by left ventricular ejection fraction (LVEF), fail to identify a large proportion of individuals at risk. A nationwide Danish study found that 44.5% of SCD cases occurred in individuals without prior cardiovascular disease, underscoring the limitations of current risk stratification approaches (5). A broader conceptualization of SCD risk stratification and modifiable risk-factor targeting is therefore warranted. Emerging evidence and contemporary cardiovascular health frameworks highlight the importance of modifiable lifestyle domains — including sleep health, physical activity, diet, psychological stress, and substance use — as determinants of cardiovascular risk and potential contributors to SCD susceptibility (7, 8). These factors are increasingly recognized to influence arrhythmogenic vulnerability through multiple biological pathways, including autonomic dysfunction, systemic inflammation, adverse cardiac remodeling, and metabolic–hormonal dysregulation (9, 10).

Despite this growing body of evidence, no recent review has systematically integrated findings across these domains within a unified mechanistic framework. Existing literature has largely addressed individual lifestyle factors in isolation — such as sleep (11), physical activity (12), or diet (13) — or focused on device-based prevention strategies (4). The present review addresses this gap by integrating evidence across five lifestyle domains within a single mechanistic framework, with emphasis on recent developments in sleep-disordered breathing, wearable-derived activity data, and AI-enabled risk prediction.

## 2 Literature search strategy

Electronic searches were performed in PubMed/MEDLINE, Web of Science, and EMBASE covering January 2003 to April 2026 in two stages, using Boolean operators combining the following MeSH and free-text terms: (sudden cardiac death OR ventricular fibrillation OR ventricular arrhythmia) AND (sleep duration OR obstructive sleep apnea OR circadian rhythm OR physical activity OR exercise OR Mediterranean diet OR psychological stress OR smoking OR alcohol OR sedentary behavior OR wearable devices OR artificial intelligence). The original search was conducted on 22 March 2025. To ensure currency at the time of submission, a supplementary verification search was performed on 28 April 2026, covering publications between April 2025 and April 2026. This update identified seven high-impact publications relevant to Mendelian randomization, exercise-associated arrhythmia, AI-enhanced electrocardiography, and wearable-device-based evidence, all of which were incorporated into the manuscript (Arora et al., Kim et al., Javed et al., Fiorina et al., van Steijn et al., Shah et al., and Lai et al.; full citations in Supplementary Material S1). Full database-specific search strings, the two-stage search timeline, and approximate screening counts are provided in Supplementary Material S1. Priority was given to prospective cohort studies, randomized controlled trials (RCTs), systematic reviews, and meta-analyses published in peer-reviewed cardiovascular and general medical journals. Study-level methodological quality, rather than journal-level metrics such as impact factor, guided inclusion, consistent with the San Francisco Declaration on Research Assessment (DORA). Clinical practice guidelines from the European Society of Cardiology (ESC), the American Heart Association/American College of Cardiology (AHA/ACC), and AHA Scientific Statements were retrieved in full. Foundational publications outside the formal search window were retained where they remain primary sources for established concepts (e.g., the original description of holiday heart syndrome by Ettinger et al., 1978; the WHO 1985 definition of SCD; and the ATRAMI baroreflex sensitivity study, 1998). Non-English publications were excluded. This introduces a potential language bias, particularly relevant given the high burden of SCD in Asian populations and the substantial body of Chinese-, Japanese-, and Korean-language epidemiological literature not indexed in English-language databases. This work is a structured narrative review rather than a systematic review or meta-analysis. Consistent with SANRA (Scale for the Assessment of Narrative Review Articles) guidance, we used pre-specified inclusion criteria (Section 2), documented a two-stage search (March 2025 + April 2026 verification), and independently verified all quantitative estimates against original full-text sources. However, we did not perform formal risk-of-bias appraisal (e.g., ROBINS-E), quantitative pooling, or PRISMA-compliant flow-diagramming, and the eligible-study universe was not exhaustively enumerated. Findings should therefore be read as a hypothesis-generating synthesis of the current evidence base rather than as pooled effect estimates, and the strength-of-evidence tiering introduced in Section 3 is intended to make this uncertainty explicit at the level of each lifestyle domain.

## 3 Pathophysiological framework: how lifestyle factors precipitate SCD

**Figure 1:**
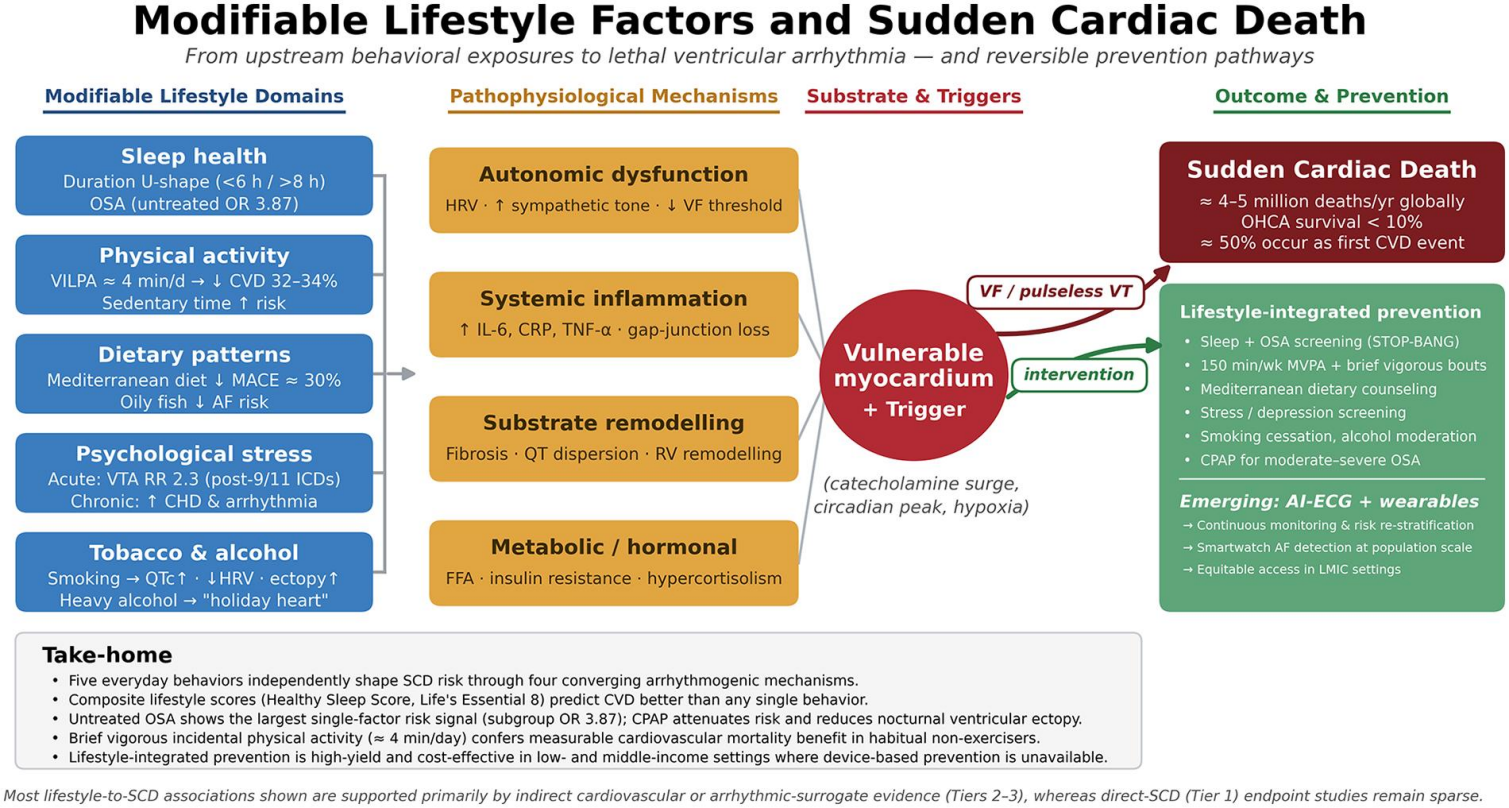
Conceptual framework linking five modifiable lifestyle domains to sudden cardiac death (SCD) and to complementary prevention pathways. Reading left to right: five modifiable lifestyle exposures (sleep health, physical activity, dietary patterns, psychological stress, tobacco and alcohol use) converge — via a single grey convergent arrow that replaces the twenty pairwise connectors of prior versions — on four pathophysiological mechanism categories (autonomic dysfunction, systemic inflammation, substrate remodelling, metabolic and hormonal dysregulation), which in turn converge on a vulnerable myocardial substrate plus an acute trigger (catecholamine surge, circadian peak, hypoxia). From this common node the framework then bifurcates into a two-arm fork: (i) an upper dark-red arm progresses through an explicit intermediate node labelled “VF/pulseless VT” and terminates in the sudden cardiac death outcome box (≈ 4–5 million deaths per year globally; out-of-hospital cardiac arrest survival < 10%; approximately 50% of events occur as the first cardiovascular manifestation), and (ii) a lower green “intervention” arm terminates in a Lifestyle-integrated prevention box that enumerates six actionable clinical measures (sleep and OSA screening, MVPA targets, Mediterranean dietary counselling, stress and depression screening, smoking cessation and alcohol moderation, and CPAP for moderate-to-severe OSA), together with three emerging AI-enhanced or wearable-device directions. A grey take-home banner summarises five headline messages. A footnote below the framework flags the evidence grading applied throughout the review: most lifestyle-to-SCD associations shown are supported primarily by indirect cardiovascular or arrhythmic-surrogate evidence (Tiers 2–3), whereas direct-SCD (Tier 1) endpoint studies remain sparse. AF, atrial fibrillation; AHI, apnoea–hypopnoea index; AI, artificial intelligence; CPAP, continuous positive airway pressure; CVD, cardiovascular disease; ECG, electrocardiography; FFA, free fatty acids; HRV, heart rate variability; MASLD, metabolic-dysfunction-associated steatotic liver disease; MVPA, moderate-to-vigorous physical activity; OHCA, out-of-hospital cardiac arrest; OSA, obstructive sleep apnoea; STOP-BANG, snoring, tiredness, observed apnoea, blood pressure, BMI, age, neck circumference, gender; VF, ventricular fibrillation; VILPA, vigorous incidental physical activity; VT, ventricular tachycardia.

Sudden cardiac death (SCD) is generally understood to result from the interaction between a vulnerable structural cardiac substrate and transient triggering factors that precipitate malignant ventricular arrhythmias (4). Coronary artery disease accounts for the majority of SCD events in adults, whereas cardiomyopathies and inherited channelopathies predominate in younger individuals. Triggering has become an established pathophysiological concept, supported by the well-characterized circadian and situational clustering of SCD events (10). This framework provides a useful basis for understanding how modifiable lifestyle factors may influence SCD risk. Within this conceptual model, five major modifiable lifestyle domains act on arrhythmogenic susceptibility through four shared mechanistic pathways (Figure 1). These pathways include autonomic dysfunction, systemic inflammation, structural and electrophysiological remodeling, and metabolic–hormonal dysregulation, which are examined in the following sections.

An important scoping clarification is in order. SCD as a clinical entity —following the WHO definition adopted in Section 1 — is agnostic to the underlying mechanism: an unexpected cardiac-cause death within a short time from symptom onset. While ventricular arrhythmias account for the majority of adjudicated SCD events, non-arrhythmic mechanisms including acute myocardial infarction with pump failure or cardiogenic shock, cardiac tamponade, massive pulmonary embolism, and aortic dissection can produce the same clinical phenotype. The mechanistic pathways and lifestyle associations reviewed in the sections that follow relate predominantly to the arrhythmogenic subset, reflecting the concentration of the modifiable-lifestyle evidence base in that domain rather than a claim of equivalence between SCD and arrhythmic death. Where a lifestyle exposure is linked primarily to acute coronary events or pump failure (e.g., stress-triggered acute myocardial infarction, smoking-related atherosclerotic burden) rather than to arrhythmogenic substrate *per se*, the intermediate mechanism is flagged in the relevant section.

Evidence tiers used throughout the review. Because the outcomes span direct SCD ascertainment, cardiovascular mortality proxies, arrhythmic surrogates, and mechanistic endpoints, we tier every quantitative claim in the sections that follow into one of four categories: Tier 1 — Direct SCD (adjudicated fatal arrhythmic events); Tier 2 — Indirect cardiovascular mortality (all-CV or ischemic mortality where SCD subtypes are not separately reported); Tier 3 — Arrhythmic surrogate (ventricular arrhythmia, atrial fibrillation, sustained VT, ICD shocks, QT prolongation, HRV disturbance); and Tier 4 — Mechanistic (autonomic, inflammatory, or substrate biomarkers without hard clinical endpoints). Tier labels are shown in-line where the effect size is first introduced and in the evidence-strength column of Table 1. The purpose is transparency: several apparently large effects (e.g., short-sleep-and-SCD associations) rest predominantly on Tier 2–3 evidence, and this should temper causal language until Tier 1 replication is available.

**Table 1:** Five modifiable lifestyle domains and sudden cardiac death risk.

Domain	Best evidence source (design + N or I^2^)	Effect estimate + 95% CI	Evidence tier (1–4)	Direction	Recommended intervention	Implementation barrier
Sleep duration & quality	Meta-analyses >3.3 M participants (Kwok, Cappuccio); European prospective Healthy Sleep Score cohort (Nambiema, EHJ 2023)	U-shape: sleep <6 h or >8 h → all-cause mortality (RR 1.14 for 9 h/night, 95% CI 1.05–1.25); HSS +1 pt → 18% lower CVD risk (HR 0.82)	Tier 2 (indirect CV mortality); Tier 3 for HRV surrogates	↑ risk at extremes	Routine sleep history + validated screening (STOP-BANG, Epworth)	Under-recognition in cardiology clinics; polysomnography access
Obstructive sleep apnea	2025 systematic review + meta-analysis (Zou et al., 12 studies, 527,069 participants; I^2^ = 79.3%)	Pooled OR 1.41 (0.91–2.16) NS; untreated subgroup OR 3.87 (1.09–13.81); AHI-graded HR 1.6→2.6	Tier 2 (pooled)/Tier 3 (untreated subgroup)	↑ risk in untreated; treatment status is a critical effect modifier	STOP-BANG screening in cardiovascular clinics; CPAP referral; treatment adherence support	PSG access; CPAP adherence rates; reimbursement variability
Physical activity — dose	UK Biobank accelerometer cohorts (Stamatakis Nat Med 2022; Strain Nat Med 2020); 2025 JAMA long-distance race analysis (Kim, 29.3 M finishers)	VILPA ≈4 min/day → 32%–34% ↓ CV mortality; U-shape at extreme endurance	Tier 2 (indirect CV mortality); Tier 3 for accelerometer-derived surrogates	↓ risk with regular moderate; ↑ risk at extreme endurance	Promote 150 min/week moderate; short vigorous bouts count	Low adherence; sedentary urban environments; time constraints
Physical activity — modality	JACC Focus Seminar 4/4 (La Gerche 2022); LIVE-HCM cohort (Lampert JAMA Cardiol 2023)	Endurance → substantial chamber remodeling; strength → modest; marked hypertrophy in strength athlete → red flag	Tier 3 for arrhythmic surrogates; Tier 4 for remodeling mechanisms	Modality-dependent; individualized assessment	Individualized pre-participation evaluation; ESC 2022 Class IIa ECG in competitive athletes	Uneven access to imaging; over-diagnosis risk in screening
Diet & metabolic exposures	PREDIMED RCT (retracted + re-published 2018); cohort meta-analyses	Mediterranean diet → MACE ↓ ∼30%; *ω*-3 EPA/DHA associated with anti-arrhythmic surrogates	Tier 2 (MACE composite; not direct SCD); Tier 4 for ion-channel mechanisms	↓ risk with adherence	Mediterranean-style counseling; olive oil, nuts, fish, vegetables	Cost; cultural acceptability; ultra-processed food environments
Psychological stress	Cohort + case-crossover designs; post-9/11 ICD data (Steinberg); narrative reviews (Steptoe & Kivimäki, Nat Rev Cardiol)	Acute stress: RR 2.3 ↑ ventricular tachyarrhythmia in ICD patients; chronic stress associated with CV mortality	Tier 3 (arrhythmic surrogate); Tier 4 (catecholamine surge mechanism)	↑ risk (acute triggers and chronic burden)	Screen for depression, social isolation, work strain; CBT and stress management referral	Stigma; limited mental health workforce; reimbursement gaps
Tobacco use	Epidemiological reviews + mechanistic studies (Prasad; D'Alessandro)	QTc prolongation, reduced HRV, ↑ ventricular ectopy; substantial excess-risk attenuation within 1 y of cessation	Tier 2 (indirect CV mortality); Tier 4 (electrophysiological mechanisms)	↑ risk; ↓ risk after cessation	Active smoking-cessation support (pharmacotherapy + counseling); tobacco control policy	Nicotine addiction; policy variability across regions
Alcohol use	Ettinger et al. (holiday heart); GBD 2016 Alcohol Collaborators; Zhao et al. systematic review; Vo/Truyen postmortem cohort (IJC HV 2025); MASLD population data (Truyen, Front CVM 2026)	Heavy drinking 28% vs. 12% in SLD-SCD; MASLD HR 1.6 (1.4–1.7) for SCD; no clear cardioprotective threshold	Tier 2 (population MASLD HR); Tier 3 (arrhythmic surrogates); Tier 4 (mechanism)	↑ risk (dose-dependent); no safe cardio-protective level	Brief interventions; minimize intake as primary public-health message; SLD screening in heavy drinkers	Alcohol industry marketing; cultural normalization; under-recognition of SLD

AHI, apnea–hypopnea index; CBT, cognitive behavioral therapy; CPAP, continuous positive airway pressure; CV, cardiovascular; CVD, cardiovascular disease; DHA, docosahexaenoic acid; EPA, eicosapentaenoic acid; ESC, European Society of Cardiology; HCM, hypertrophic cardiomyopathy; HR, hazard ratio; HRV, heart rate variability; HSS, Healthy Sleep Score; ICD, implantable cardioverter-defibrillator; MACE, major adverse cardiovascular events; MASLD, metabolic-dysfunction-associated steatotic liver disease; OR, odds ratio; OSA, obstructive sleep apnea; PSG, polysomnography; QTc, corrected QT interval; RCT, randomized controlled trial; RR, relative risk; SCD, sudden cardiac death; SLD, steatotic liver disease; STOP-BANG, snoring–tiredness–observed apnea–blood pressure–BMI–age–neck circumference–gender; VILPA, vigorous incidental physical activity.

### 3.1 Autonomic dysfunction and sympathovagal imbalance

Reduced heart rate variability (HRV) is a well-established independent predictor of sudden cardiac death (SCD) and cardiovascular mortality. The landmark ATRAMI study showed that a 24-hour SDNN below 70 ms was associated with a 3.2-fold multivariate-adjusted relative risk of cardiac death (95% CI 1.42–7.36), independent of left ventricular ejection fraction and ventricular ectopy (15). Cardiac electrophysiology depends on a dynamic balance between sympathetic and vagal input. When vagal tone decreases, sympathetic dominance lowers the ventricular fibrillation threshold, shortens effective refractory periods, and increases susceptibility to triggered arrhythmias through catecholamine-mediated calcium overload and early afterdepolarisations (16, 17). Several common lifestyle exposures — including sleep deprivation, chronic psychological stress, smoking, and physical inactivity — are associated with reduced HRV and elevated resting sympathetic tone. The clinical relevance of HRV as a predictor of SCD has been extensively demonstrated in post-myocardial infarction and heart failure populations. Importantly, HRV represents a potentially modifiable target: aerobic exercise training, cognitive behavioral stress reduction, smoking cessation, and improved sleep quality have been shown to enhance vagal tone over weeks to months, consistent with findings from interventional and longitudinal studies. This positions autonomic imbalance not only as a marker of risk but also as a modifiable intermediate phenotype linking lifestyle exposures to arrhythmic outcomes.

### 3.2 Systemic inflammation and endothelial dysfunction

Chronic low-grade inflammation contributes to arrhythmogenesis. The CANTOS trial provided important causal evidence: interleukin-1β inhibition with canakinumab (150 mg every three months) reduced recurrent major cardiovascular events by 15% (HR 0.85, 95% CI 0.74–0.98) among 10,061 patients with prior myocardial infarction and high-sensitivity C-reactive protein ≥2 mg/L, independent of lipid lowering (18). Inflammatory mediators such as interleukin-6, C-reactive protein, and tumor necrosis factor-α promote adverse cardiac remodeling, accelerate myocardial fibrosis, and impair gap junction function, thereby facilitating re-entrant arrhythmias. Smoking, excessive alcohol consumption, sedentary behavior, and pro-inflammatory dietary patterns are established lifestyle contributors to systemic inflammation (12, 17).

Although trials specifically powered for SCD endpoints remain limited, converging mechanistic and epidemiological evidence supports inflammation as a key shared pathway through which several lifestyle exposures — particularly diet, smoking, sleep disruption, and sedentary behavior — may influence arrhythmic risk. Measurement of high-sensitivity CRP may help identify individuals at higher cardiovascular risk who could benefit from targeted lifestyle interventions. This highlights inflammation as a mechanistic bridge linking lifestyle exposures to arrhythmic outcomes.

### 3.3 Arrhythmic substrate remodeling

Subclinical myocardial substrate remodeling — including replacement fibrosis and diffuse interstitial fibrosis, both of which are now detectable with cardiac magnetic resonance imaging using late gadolinium enhancement and T1 mapping — is a major independent prognostic factor across diverse cardiomyopathies (19). Several lifestyle exposures can directly influence these structural and electrophysiological properties. Intermittent hypoxia in untreated obstructive sleep apnea (OSA) has been associated with QT interval prolongation and increased repolarization dispersion, which may increase susceptibility to ventricular arrhythmias (9). Extreme endurance training may accelerate right ventricular remodeling and fibrosis in genetically predisposed individuals, particularly those with subclinical arrhythmogenic substrates (20). Chronic psychological stress contributes through catecholamine-mediated cardiomyocyte injury, promoting adverse ventricular remodeling over time (17).

Lifestyle-driven substrate changes are typically subtle, cumulative, and clinically silent until an arrhythmic trigger precipitates SCD. The temporal gap between chronic exposure and acute events poses a diagnostic challenge, as current risk models capture substrate alterations only indirectly. At the same time, it presents an opportunity: AI-enhanced ECG and imaging analytics may potentially extract latent risk signatures not readily detectable by conventional methods (19, 21).

Progress in translating substrate biology into individualized prediction is illustrated by the VFRisk score, a multivariable clinical prediction model originally developed in the Oregon Sudden Unexpected Death Study (C-statistic 0.808) and replicated in Ventura County (C-statistic 0.782). External validation in a nested case-referent analysis within the Framingham Heart Study achieved a C-statistic of 0.711 (95% CI 0.656–0.767) for sudden cardiac death, with stable performance across age (≤65 vs. >65 years) and sex strata (62). Although discrimination is modest relative to the derivation cohorts — likely reflecting the older, predominantly European-ancestry FHS population and the absence of some model inputs such as LVH — this line of work establishes a plausible bridge between the mechanistic pathways summarized in Sections 3.1–3.4 and pragmatic risk-stratification tools that could, in future, be linked to modifiable exposures. (Evidence tier: 1 for the SCD outcome; 3 for the score's operating characteristics.).

### 3.4 Metabolic and hormonal dysregulation

Disordered glucose and lipid metabolism contributes to an arrhythmogenic milieu. A recent meta-review by Wang et al., integrating observational and Mendelian randomization evidence, summarises associations between sleep duration and cardiovascular outcomes and highlights potential links between sleep-related metabolic disturbances and incident cardiovascular disease (22). As this work synthesizes multiple MR studies rather than presenting a single primary analysis, the strength of causal inference varies across the included estimates. The downstream physiology is multi-layered. Elevated free fatty acids may impair cardiac ion channel function. Insulin resistance is associated with increased sympathetic activation. Hypercortisolemia, partly driven by chronic sleep restriction, may enhance myocardial sensitivity to catecholamine-induced arrhythmias (23). Poor dietary quality, sedentary behavior, and chronic sleep restriction lie upstream of these pathways and amplify metabolic-arrhythmic risk through their combined effects on insulin signaling and inflammatory tone.

These four mechanistic axes do not operate in isolation. A single lifestyle exposure — such as chronic sleep restriction — can simultaneously suppress HRV, elevate inflammatory markers (e.g., IL-6 and CRP), accelerate atherogenesis, and induce insulin resistance, thereby exerting convergent pressure on the arrhythmic substrate. This multiplicity helps explain why composite lifestyle scores [such as Life's Essential 8 (8) and the Healthy Sleep Score (24)] often demonstrate stronger predictive performance than any single behavior, and why interventions targeting multiple domains in parallel may yield greater reductions in SCD risk than single-factor approaches.

## 4 Sleep health and sudden cardiac death

Sleep is increasingly recognized as a core pillar of cardiovascular health, formally incorporated into the AHA Life's Essential 8 framework in 2022 (8). Evidence from multiple domains — epidemiological cohorts, mechanistic studies, and intervention trials — demonstrates that sleep disturbance contributes to SCD risk through the autonomic, inflammatory, and arrhythmogenic pathways described in Section 3.

### 4.1 Sleep duration: a U-shaped relationship with cardiovascular mortality

Sleep duration is associated with cardiovascular outcomes in a U-shaped pattern. In a meta-analysis of more than 3.3 million participants, Kwok et al. reported that self-reported sleep of 9 h per night was associated with an increased risk of all-cause mortality (relative risk [RR] 1.14, 95% CI 1.05–1.25), with higher risks observed at longer durations (25). Short sleep duration is similarly associated with adverse outcomes, although effect sizes vary by endpoint. Cappuccio et al. observed a 48% increase in coronary heart disease incidence and mortality among short sleepers (RR 1.48, 95% CI 1.22–1.80) and a 15% increase for stroke (RR 1.15, 95% CI 1.00–1.31), whereas the pooled estimate for total cardiovascular disease was not statistically significant (RR 1.03, 95% CI 0.93–1.15), suggesting endpoint-specific heterogeneity rather than a uniform dose–response relationship (26). Huang et al., pooling 71 cohorts and 3.8 million participants, further characterized this association as non-linear, with risk minimized at approximately 7.5 h per night (*p* for non-linearity <0.0001) (27). Mendelian randomization (MR) studies have begun to explore potential causal relationships. A 2025 MR analysis by Arora et al., using UK Biobank and HUNT2 data, reported that genetically predicted insomnia (HR 1.14, 95% CI 1.07–1.21) and short sleep duration (HR 1.14, 95% CI 1.04–1.26) were associated with incident atrial fibrillation. However, no synergistic effect between combined sleep traits was observed, and replication in the HUNT2 cohort showed attenuation of effect estimates (28). A meta-review integrating observational and MR evidence is broadly consistent with a causal interpretation, although, as a synthesis rather than a primary MR analysis, it does not independently establish causality (22). Taken together, these findings suggest that aberrant sleep duration and quality may contribute causally to atrial-arrhythmic risk, while exposure-specific MR analyses for ventricular arrhythmias and SCD endpoints remain limited.

Several mechanisms plausibly link short sleep duration to heightened arrhythmia susceptibility. Sympathovagal balance shifts toward sympathetic dominance, with nocturnal HRV suppression detectable even before overt clinical disease. Reduced slow-wave sleep is associated with increased pro-inflammatory cytokines, and chronic activation of the hypothalamic–pituitary–adrenal axis sustains elevated daytime cortisol levels. In parallel, impaired glucose metabolism contributes to the development of an arrhythmogenic metabolic substrate over time (11, 29). In contrast, long sleep duration may reflect underlying illness or fragmented sleep architecture rather than restorative sleep and has also been associated with increased systemic inflammation (11). Because short sleep clusters phenotypically with depressive and anxiety symptoms, and because non-cardiovascular comorbidities collectively account for approximately 40% of SCD events, sleep-duration effects should be interpreted alongside adjacent psychiatric risk. A recent mini-review of non-cardiovascular contributors to SCD reports that depression is associated with an approximately 1.6-fold increased SCD hazard (HR 1.6, 95% CI 1.4–1.9 in pooled analysis, rising to ∼2.7 in severe or treatment-resistant subgroups), and anxiety with an approximately 1.6- to 1.9-fold increase depending on the anxiety scale used (63). Two non-exclusive interpretations warrant consideration. Under a confounding framing, mood-related comorbidity independently elevates SCD risk and inflates sleep-duration effect estimates via residual confounding. Under a manifestation framing, because sleep disturbance is one of the nine DSM-5 diagnostic criteria for major depressive disorder, the sleep–SCD association may partly reflect the effect of underlying mood pathology channelled through sleep as a phenotypic marker. Distinguishing these two accounts — and quantifying any mediated proportion — will require adequately powered mediation analyses that current studies have not attempted. We accordingly treat isolated sleep-duration risk estimates as an upper bound and interpret sleep-mediated SCD risk in the context of adjacent psychiatric comorbidity. (Evidence tier: 2 for the pooled comorbidity estimates; 4 for the shared autonomic-dysregulation mechanism.).

### 4.2 Sleep quality, regularity, and circadian rhythm disruption

Beyond duration, sleep quality and temporal regularity represent independent determinants of cardiovascular risk. In a 2023 European Heart Journal study, Nambiema et al. analyzed two European prospective cohorts and constructed a composite Healthy Sleep Score (HSS, range 0–5) incorporating five domains: chronotype, duration, insomnia symptoms, sleep apnea, and daytime sleepiness. The HSS predicted incident cardiovascular events independently of individual sleep components. Each one-point increase in baseline HSS was associated with an 18% lower CVD risk (HR 0.82, 95% CI 0.76–0.89), while improvement in HSS over 2–5 years was associated with a 16% risk reduction (HR 0.84, 95% CI 0.73–0.97) (24). These findings highlight that dynamic improvements in sleep health, rather than a single cross-sectional measure, may have prognostic significance.

Circadian rhythm disruption — including shift work, social jetlag, and irregular sleep–wake timing — has been associated with increased SCD risk. The well-documented morning peak in SCD events (06:00–12:00) likely reflects concurrent increases in sympathetic activity, platelet aggregability, and cortisol secretion during the transition from sleep to wakefulness (30). A secondary peak during the early morning hours (00:00–06:00) has been linked to nocturnal sleep disturbance, autonomic dysregulation, and obstructive sleep apnea (9, 10).

### 4.3 Obstructive sleep apnea: a clinically important modifiable risk factor

Obstructive sleep apnea (OSA) is a clinically important and potentially modifiable risk factor for sudden cardiac death (SCD) that remains underrecognized in routine cardiovascular risk assessment. The strength of the population-level association, however, remains debated, with recent meta-analytic evidence demonstrating substantial heterogeneity. OSA is characterized by recurrent upper airway collapse during sleep, leading to cyclical intermittent hypoxia, intrathoracic pressure swings, and repeated arousals. These physiological perturbations are associated with downstream effects including oxidative stress, QT interval prolongation with increased repolarization dispersion, structural cardiac remodeling, and pronounced nocturnal sympathetic activation (9). A large prospective cohort study of 10,701 adults undergoing diagnostic polysomnography demonstrated that OSA severity, quantified by apnea–hypopnea index and nocturnal oxygen desaturation, was independently associated with incident SCD over a mean follow-up of 5.3 years (31).

Meta-analytic evidence for the OSA–SCD relationship is heterogeneous: the pooled random-effects estimate does not reach significance (OR 1.41, 95% CI 0.91–2.16, I^2^ = 79.3%), but restricting to untreated OSA yields a markedly elevated risk (OR 3.87, 95% CI 1.09–13.81). Both estimates are presented here to avoid selective reporting, and the practical implication — that treatment status is a critical effect modifier — is developed in Section 7.2. The most comprehensive recent evidence synthesis is provided by the 2025 systematic review and meta-analysis by Zou et al., which included 12 studies and 527,069 participants. The pooled estimate suggested a non-significant association between OSA and SCD (OR 1.41, 95% CI 0.91–2.16; *p* = 0.12), with substantial heterogeneity (I^2^ = 79.3%). Subgroup analyses, however, indicated that untreated OSA was associated with a higher estimated risk (OR 3.87, 95% CI 1.09–13.81), whereas no significant association was observed among CPAP-treated individuals. Sensitivity analyses suggested moderate robustness, although potential publication bias could not be excluded (14).

These findings warrant cautious interpretation. The non-significant overall estimate and high heterogeneity likely reflect differences in OSA definitions, SCD ascertainment, and study populations. The attenuation of risk estimates in treated populations and the elevated risk observed in untreated individuals provide indirect support for a potential causal relationship, although definitive conclusions cannot be drawn from current evidence. Interventional and mechanistic studies offer further insight. In a randomized controlled trial of heart failure patients with OSA, continuous positive airway pressure (CPAP) therapy reduced ventricular premature beats by 58%, accompanied by reductions in sympathetic activity (32). In a prospective study of patients with moderate-to-severe OSA, clinically significant nocturnal rhythm disturbances were abolished in 7 of 8 patients following CPAP therapy (33). While these studies are not designed to evaluate SCD endpoints, they provide mechanistic support for the hypothesis that effective OSA treatment may reduce arrhythmic burden. Taken together, the available evidence supports the view that OSA represents a potentially modifiable contributor to arrhythmic risk, particularly in untreated individuals. From a clinical perspective, heightened awareness, appropriate screening, and effective treatment of OSA may form an important component of integrated strategies for SCD risk reduction.

Mechanistically, chronic intermittent hypoxia in untreated OSA plausibly links to SCD through an OSA → atrial fibrillation/hypertension → arrhythmic substrate cascade. This substrate-mediated component is compatible with recent multivariable prediction work incorporating substrate-related electrocardiographic features (62). At the population level, the OSA–SCD gradient scales with disease severity: HR 1.6 (95% CI 1.1–2.2) for moderate OSA (AHI >20) and RR 2.6 (95% CI 1.5–4.4) for severe OSA (AHI ≥40) in prospective polysomnography-based follow-up, with parallel gradients for COPD (HR 1.3; frequent exacerbations HR 2.4–5.4) (63). This pathway framing is compatible with, but does not itself demonstrate, causality. (Evidence tier: 2 for the exposure–response gradient; 3 for the arrhythmic surrogate; 4 for the intermittent-hypoxia mechanism.).

## 5 Physical activity, exercise, and sudden cardiac death

The relationship between physical activity and SCD is characterized by a paradox: while habitual moderate-intensity exercise is among the well-established cardioprotective interventions, vigorous exercise transiently increases the risk of acute cardiac events — particularly in individuals with undetected structural heart disease. Understanding this U-shaped dose-response relationship is essential for clinical counseling.

### 5.1 Cardioprotective mechanisms of regular aerobic exercise

Long-term regular moderate-intensity aerobic exercise produces a broad range of cardioprotective adaptations. Resting heart rate declines, vagal tone increases, and heart rate variability (HRV) improves, collectively contributing to enhanced electrical stability and potentially increasing the ventricular fibrillation threshold. Coronary endothelial function improves, and collateral circulation may expand. Sympathoadrenal responses to physiological stressors become attenuated, and circulating inflammatory markers are reduced. In parallel, several favorable metabolic adaptations occur, including reduced visceral adiposity, improved insulin sensitivity, and a more favorable lipid profile (12, 34). At the population level, achieving the World Health Organization recommendation of at least 150 min per week of moderate-intensity aerobic activity has been associated with substantial reductions in cardiovascular risk. In a meta-analysis of 36 studies comprising 3.4 million participants, meeting this threshold was associated with a 23% reduction in cardiovascular mortality and a 17% reduction in incident cardiovascular disease (35).

### 5.2 The U-shaped dose–response: from protection to risk

At both extremes of the physical activity spectrum, SCD risk may increase. Sedentary behavior is independently associated with higher cardiovascular mortality through adverse metabolic effects, increased thrombogenicity, and impaired autonomic regulation. Importantly, the adverse effects of prolonged sitting are not fully offset by achieving recommended levels of physical activity during other parts of the day (36).

At the higher-intensity end of the spectrum, contemporary epidemiological data refine this relationship. A 2025 JAMA study by Kim et al. analyzed 29.3 million long-distance race finishers between 2010 and 2023 and identified 176 cardiac arrests, corresponding to an incidence of 0.54 per 100,000 participants — similar to the 2000–2009 baseline (0.60 per 100,000). Notably, survival improved substantially, with the case fatality rate declining from 71% to 34%, and the incidence of cardiac death decreasing to 0.20 per 100,000 (95% CI 0.15–0.26). Risk was higher among men (1.12 vs. 0.19 per 100,000), in marathon versus half-marathon events (1.04 vs. 0.47 per 100,000), and in cases attributable to coronary artery disease (52% of events with a definitive cause), providing an updated estimate of contemporary exercise-related SCD epidemiology (37).

At the opposite extreme, long-term high-volume endurance training has been associated with a paradoxical increase in SCD risk, likely mediated by pathological cardiac remodeling in susceptible individuals. Extreme endurance exercise may promote right ventricular fibrosis and arrhythmogenic substrate formation, particularly in those with underlying or subclinical arrhythmogenic right ventricular cardiomyopathy (ARVC) (20). The 2025 VENTOUX cohort (Javed et al., Circulation: Cardiovascular Imaging) extends these observations. Among 106 male veteran endurance athletes aged ≥50 years (≥10 h/week training for ≥15 years) followed for a median of 720 days, focal myocardial fibrosis was detected by cardiac MRI in 47.2% of participants, and ventricular arrhythmias were identified in 21.7% during prolonged monitoring (sustained ventricular tachycardia in 2.8%, non-sustained in 18.9%). The presence of fibrosis was associated with an increased risk of incident ventricular arrhythmia (hazard ratio 4.7, 95% CI 1.8–12.8; *p* = 0.002), independent of left ventricular dilatation (38). In addition, the risk of atrial fibrillation is elevated approximately 3–5-fold in endurance athletes compared with age-matched controls (20). In susceptible individuals, atrial arrhythmias may act as triggers that facilitate the initiation of ventricular arrhythmias.

The upper limb of the U-shape must be interpreted through the modality of the underlying exercise, not total volume alone. Endurance-trained athletes develop eccentric hypertrophy driven by chronic volume overload, characterised by balanced left- and right-ventricular chamber enlargement with proportionate wall thickness, whereas strength- and isometric-trained athletes develop concentric hypertrophy driven by pressure overload, typically with modest chamber enlargement (64). Regarding which pattern carries the greater sudden cardiac death signal: eccentric remodelling has the stronger empirical association with adverse arrhythmic outcomes — the VENTOUX cohort in veteran endurance athletes documented ventricular arrhythmias in 21.7% and a fibrosis-associated hazard ratio of 4.7 for incident ventricular arrhythmia (see above) — mediated in part by right-ventricular fibrosis and atrial-arrhythmia triggers. Concentric hypertrophy in isolation is generally considered physiological, but marked disproportionate hypertrophy in a strength-trained athlete should raise suspicion of underlying hypertrophic cardiomyopathy or other pathological substrate, in which case SCD risk is driven by the underlying disease rather than by exercise itself. Both remodelling patterns can therefore be associated with elevated SCD risk, but via distinct mechanisms and susceptibility profiles. We accordingly avoid a single “safe upper limit” recommendation and endorse individualised assessment aligned with contemporary pre-participation screening consensus (Section 7.3). (Evidence tier: 3 for arrhythmic surrogates in athlete cohorts; 4 for the remodelling mechanisms.).

### 5.3 Vigorous incidental physical activity and wearable device evidence

Large accelerometer-based cohort studies have substantially reshaped the understanding of brief vigorous activity, introducing the concept of vigorous incidental physical activity (VILPA): short, unstructured bursts of vigorous movement embedded in daily life. In a 2022 Nature Medicine analysis, Stamatakis et al. followed 25,241 habitual non-exercisers in the UK Biobank for a mean of 6.9 years using accelerometer-based exposure measurement. At the sample median VILPA dose of approximately 4.4 min per day (corresponding to three bouts of 1–2 min), participants demonstrated a 26%–30% lower risk of all-cause and cancer mortality and a 32%–34% lower risk of cardiovascular mortality compared with those with no VILPA (39).

The dose–response relationship was monotonic, with greater reductions observed at higher levels of exposure; the highest modeled levels were associated with up to a 38%–40% reduction in all-cause and cancer mortality and up to a 48%–49% reduction in cardiovascular mortality (39). In this review, the median-dose estimate (∼4.4 min/day, corresponding to a 32%–34% reduction in CVD mortality) is emphasized as a clinically achievable reference point for habitual non-exercisers, whereas higher-dose estimates represent the upper bound of modeled benefit. Although these effect sizes should be interpreted within the context of a non-exercising baseline population, they suggest potential clinical relevance for individuals unable or unwilling to engage in structured exercise programs.

A complementary UK Biobank study by Strain et al., also published in Nature Medicine, demonstrated that higher physical activity energy expenditure (PAEE), objectively measured using wrist-worn accelerometers in 96,476 participants, was associated with a lower hazard of all-cause mortality across the full spectrum of activity levels, supporting the principle that incremental increases in activity volume are associated with cardiovascular benefit (40).

### 5.4 Exercise-associated SCD: high-risk populations and pre-participation screening

The absolute risk of exercise-associated SCD in the general population is low, estimated at approximately 1 in 15,000–18,000 vigorous exercisers per year based on the Rhode Island jogging study and the Seattle cardiac arrest study (41), but is substantially higher in individuals with undetected structural or electrical cardiac disease. A 2024 JACC State-of-the-Art Review reported an incidence of approximately 1 in 50,000–80,000 athlete-years, with significant variation by sport, sex, and ethnicity (16). Complementary population-level prospective data from Bagnall et al. in the New England Journal of Medicine reported an SCD incidence of approximately 1.3 per 100,000 person-years in Australians aged 1–35 years, with approximately 40% of cases remaining unexplained after protocol-driven post-mortem investigation — underscoring both the low absolute incidence in the young and the residual burden attributable to primary electrical disorders and unclassified cardiomyopathies (6). The most commonly implicated underlying conditions — including hypertrophic cardiomyopathy (HCM), arrhythmogenic right ventricular cardiomyopathy (ARVC), anomalous coronary artery origin, long QT syndrome, and Brugada syndrome — frequently remain clinically silent until the first presentation.

The LIVE prospective cohort study by Lampert et al. evaluated the relationship between vigorous exercise and ventricular arrhythmias in 1,660 patients with HCM over a median follow-up of nearly three years. In contrast to historical concerns, participation in vigorous-intensity exercise was not associated with an increased risk of ventricular arrhythmia events or mortality compared with non-vigorous activity, supporting a more individualized approach to exercise recommendations in this population (42).

Pre-participation cardiovascular evaluation, including 12-lead electrocardiography, should be considered (Class IIa recommendation) according to the 2022 ESC Guidelines for competitive athletes (4). In contrast, the AHA/ACC model relies primarily on history and physical examination without routine ECG screening, which has been associated with lower sensitivity for detecting certain high-risk conditions. Advances in artificial intelligence–enhanced ECG analysis may help address this transatlantic screening debate by improving specificity and reducing false-positive rates that have historically limited large-scale screening programs (21).

## 6 Other modifiable lifestyle factors

### 6.1 Tobacco smoking

Tobacco smoking is among the most well-established modifiable risk factors for sudden cardiac death (SCD). Smoking contributes to SCD pathogenesis through multiple arrhythmogenic mechanisms. Nicotine activates sympathetic ganglia and promotes catecholamine release, carbon monoxide impairs myocardial oxygen delivery, and combustion products such as acrolein induce endothelial injury, oxidative stress, and platelet hyper-aggregability (43).

At the electrophysiological level, smoking has been associated with QTc interval prolongation, impaired cardiac autonomic regulation as reflected by reduced heart rate variability (HRV), and increased ventricular ectopy, all of which may increase susceptibility to malignant arrhythmias (44). Smoking cessation is associated with progressive cardioprotective benefit, with substantial attenuation of excess cardiovascular risk observed within the first year after cessation (43).

### 6.2 Alcohol consumption

The cardiovascular effects of alcohol are strongly dose-dependent. Acute heavy alcohol intake can precipitate the “holiday heart syndrome,” first described by Ettinger et al. in 1978, characterized by atrial fibrillation occurring in otherwise non-alcoholic individuals following episodes of excessive drinking (45). Proposed mechanisms include autonomic imbalance, direct myocardial toxicity, electrolyte disturbances (particularly hypomagnesemia and hypokalemia), and sympathetic rebound. In susceptible individuals, atrial arrhythmias may facilitate the development of ventricular arrhythmias.

Chronic heavy alcohol use has been associated with alcoholic cardiomyopathy, a dilated cardiomyopathy phenotype with an elevated risk of malignant ventricular arrhythmia (46).

A distinct — and, until recently, under-appreciated — pathway links heavy alcohol use to SCD via fatty liver disease. In a postmortem cohort of 162 adjudicated SCD cases with histological liver assessment (101 with steatotic liver disease, 61 without), heavy drinking was documented in 28% of SCD decedents with SLD versus 12% without (*p* = 0.008), alongside higher BMI (31.6 ± 7.6 vs. 26.7 ± 5.7, *p* < 0.001) and characteristic biochemical differences (65). Consistent with this, at population scale metabolic-dysfunction-associated steatotic liver disease (MASLD) carries an HR of 1.6 (95% CI 1.4–1.7) for SCD (63). Together, these findings support an alcohol → SLD/MASLD → metabolic-inflammatory milieu → arrhythmic substrate cascade that extends the mechanistic account beyond acute holiday-heart physiology. Single-center design and modest cohort size constrain generalizability. (Evidence tier: 2–3).

The cardiovascular evidence base for alcohol has evolved substantially in recent years. The Global Burden of Disease 2016 Alcohol Collaborators reported that the level of alcohol consumption associated with the lowest overall health risk is close to zero standard drinks per week (47). A 2023 WHO European Office viewpoint published in *The Lancet Public Health* similarly concluded that no level of alcohol consumption can be considered completely safe when all health outcomes are taken into account (48).

Bias-corrected observational analyses have further challenged the traditional J-shaped curve hypothesis. A large systematic review and meta-analysis by Zhao et al., including 107 cohort studies and 4.8 million participants, attributed the apparent protective effect of light-to-moderate drinking largely to methodological biases, including “sick-quitter” effects, heterogeneity among abstainers, and residual socioeconomic confounding (49).

Taken together, contemporary evidence suggests that lower levels of alcohol consumption are associated with reduced overall health risk, and that minimizing alcohol intake may be a more appropriate public health message than earlier moderation-based frameworks.

### 6.3 Psychological stress and emotional triggers

Acute and chronic psychological stress contributes to SCD risk through neurohormonal mechanisms. Intense emotional stress triggers a catecholamine surge that can induce transient myocardial ischemia, promote abnormal automaticity and triggered activity, and lower the threshold for ventricular fibrillation. These mechanisms underlie the well-documented increases in SCD incidence following acute stressors such as natural disasters, major sporting events, and bereavement.

Takotsubo (stress) cardiomyopathy represents a striking clinical manifestation of this pathway. Characterized by transient left ventricular apical ballooning in response to intense emotional or physical stress, it has been associated with a non-negligible mortality risk, with annual mortality rates reported up to 5.6% in the International Takotsubo Registry (50).

Chronic psychological stress has also been linked to adverse cardiovascular outcomes. A comprehensive review by Steptoe and Kivimäki in *Nature Reviews Cardiology* reported that work-related stress, social isolation, depression, and adverse psychosocial environments are associated with persistently elevated sympathetic activity, reduced heart rate variability (HRV), and systemic inflammation — factors that may progressively increase arrhythmic susceptibility over time (17).

The acute arrhythmogenic impact of extreme psychological stress is illustrated by studies in implantable cardioverter-defibrillator (ICD) populations. Following the September 11, 2001 World Trade Center attacks, Steinberg et al. observed a more than twofold increase (RR 2.3) in life-threatening ventricular arrhythmias in the month after the event (51), supporting a role for acute stress as a potential trigger in susceptible individuals.

### 6.4 Dietary patterns

Dietary quality may influence SCD risk through multiple pathways, including modulation of systemic inflammation, lipid metabolism, blood pressure, glycemic control, and electrophysiological stability. The Mediterranean dietary pattern — characterized by high intake of olive oil, fruits, vegetables, legumes, whole grains, fish, and nuts — has the most consistent evidence for cardiovascular risk reduction.

The PREDIMED randomized controlled trial demonstrated that a Mediterranean diet supplemented with either extra-virgin olive oil or mixed nuts was associated with an approximately 30% reduction in major cardiovascular events compared with a low-fat control diet among 7,447 high-risk adults followed for a median of 4.8 years (13).

The original 2013 PREDIMED report was retracted and republished in 2018 after randomization irregularities at some recruitment sites; sensitivity analyses on the verifiable subset (∼79% of participants) yielded largely unchanged effect estimates. PREDIMED findings are therefore interpreted here as supportive rather than definitive evidence. Notably, this evidence base — including PREDIMED and its supporting meta-analyses — speaks primarily to composite major-adverse-cardiovascular-event outcomes rather than to adjudicated sudden cardiac death; SCD-specific confirmation from adequately powered dietary intervention trials remains outstanding, and the anti-arrhythmic inference drawn here is therefore best characterised as Tier 2 (indirect cardiovascular mortality) evidence rather than Tier 1 (direct SCD).

Long-chain omega-3 polyunsaturated fatty acids (EPA and DHA) may exert anti-arrhythmic effects through modulation of cardiac ion channels, reduction of ventricular repolarization heterogeneity, and anti-inflammatory actions. These mechanisms may partly explain the observed associations between fish consumption and reduced incidence of atrial fibrillation (52).

### 6.5 Sedentary behavior

Sedentary behavior — defined as waking behavior in a sitting or reclining posture with low energy expenditure — is an independent cardiovascular risk factor that is not fully offset by meeting recommended levels of physical activity.

A systematic review and meta-analysis by Biswas et al. reported that each additional hour of daily sedentary time was associated with a 2%–3% increase in all-cause and cardiovascular mortality after adjustment for physical activity levels, with the greatest risk observed among individuals with the lowest overall activity (36).

Interventions aimed at interrupting prolonged sedentary time — such as brief activity breaks of 2–3 min of light walking per hour — have been associated with improvements in postprandial glycemia and vascular function, suggesting a potential role for behavioral modification in cardiovascular risk reduction.

## 7 Prevention strategies and clinical implications

The convergence of epidemiological evidence, mechanistic insight, and emerging digital health technologies provides a reasonable foundation for a comprehensive, lifestyle-integrated approach to SCD primary prevention. Table 1 summarises the evidence base, key effect estimates, recommended interventions, and implementation barriers across the five lifestyle domains discussed in this review.

### 7.1 Integrated lifestyle risk assessment in clinical practice

Current SCD risk stratification is dominated by LVEF-based models that identify only a minority of future SCD victims. An integrated lifestyle risk assessment — encompassing sleep health, physical activity, dietary quality, psychological wellbeing, and substance use — should be incorporated as a standard component of cardiovascular risk evaluation. The AHA Life's Essential 8 framework, which formally incorporates sleep health alongside blood pressure, blood lipids, blood glucose, body weight, smoking, diet, and physical activity, provides a validated composite metric for cardiovascular health scoring (8). A structured questionnaire addressing each of these eight domains — requiring only 5–10 min to administer — could identify patients warranting targeted lifestyle intervention.

### 7.2 Obstructive sleep apnea screening and management

OSA is a high-yield screening target for reducing modifiable arrhythmic risk in cardiovascular populations. Three considerations support this position: high prevalence among at-risk cardiovascular populations, the markedly elevated SCD risk in untreated patients (subgroup OR 3.87), and the availability of an effective therapy in CPAP. Practical screening tools — the Epworth Sleepiness Scale, the STOP-BANG questionnaire, and overnight pulse oximetry — are widely accessible; suspected or confirmed moderate-to-severe disease should proceed to formal polysomnography. Two contemporary AHA scientific statements support this approach. The 2021 AHA Scientific Statement on OSA and Cardiovascular Disease recommends evaluation for sleep apnea in patients with ventricular tachycardia and in survivors of sudden cardiac death where OSA is suspected (53); the 2022 AHA Scientific Statement on Sleep-Disordered Breathing and Cardiac Arrhythmias extends this by endorsing SDB screening as best practice in patients with ventricular tachyarrhythmias and those at risk for SCD (54). A comprehensive JAMA review further supports the observation that positive airway pressure therapy lowers blood pressure, with the largest benefit in resistant hypertension (55). At a practical level, cardiovascular clinicians should ask about snoring, witnessed apneas, and non-restorative sleep at every cardiovascular risk consultation.

### 7.3 Pre-participation cardiovascular evaluation

Pre-participation cardiovascular evaluation may help identify individuals at increased risk of exercise-associated sudden cardiac death (SCD), particularly among those planning to engage in vigorous physical activity (4). Assessment should include a focused clinical history addressing unexplained syncope, exertional palpitations, premature SCD in first-degree relatives (<50 years), and known cardiomyopathy or channelopathy.

In competitive athletes, a 12-lead electrocardiogram (ECG) may be considered (Class IIa recommendation according to the 2022 ESC Guidelines), and ECG screening may also be reasonable in individuals aged ≥35 years initiating high-intensity training (4). Identification of underlying conditions such as hypertrophic cardiomyopathy (HCM), arrhythmogenic right ventricular cardiomyopathy (ARVC), Brugada syndrome, long QT syndrome, or anomalous coronary artery origin may allow timely risk stratification and individualized management, including consideration of implantable cardioverter-defibrillator (ICD) therapy, exercise modification, and pharmacological intervention (16).

### 7.4 Emerging technologies: artificial intelligence and wearable devices

Artificial intelligence (AI)–enhanced electrocardiography has been proposed as an emerging complement to conventional SCD risk stratification. Reported machine-learning models integrating ECG features with clinical data have shown moderate discrimination for future arrhythmic events in single-center cohorts (21), but external validation across diverse populations remains limited and no lifestyle-modification-based prospective evaluation has yet been conducted.

Consumer-grade wearable devices may offer additional opportunities for early detection of arrhythmias. The Apple Heart Study demonstrated the feasibility of smartwatch-based photoplethysmography (PPG) algorithms for identifying previously undiagnosed atrial fibrillation in a large population; however, important limitations include low rates of confirmatory ECG follow-up, potential selection bias, and uncertain false-positive rates in real-world settings (56).

Recent randomized evidence further informs this field. The EQUAL trial (van Steijn et al., JACC 2026) evaluated Apple Watch–based monitoring in 437 older individuals at elevated stroke risk and reported higher detection rates of incident atrial fibrillation compared with usual care (9.6% vs. 2.3%; HR 4.40, 95% CI 1.66–11.66). While these findings suggest potential benefit in targeted high-risk populations, confirmation in larger and more diverse cohorts is warranted (57). It is worth emphasising that detection of previously undiagnosed atrial fibrillation is not equivalent to sudden cardiac death prevention; whether earlier AF detection through consumer-grade wearables translates into reduced SCD incidence — as opposed to reduced stroke incidence, for which the causal chain is better established — remains to be tested prospectively with SCD as the primary endpoint.

Advances in wearable-based detection of critical events are also emerging. A 2025 study in *Nature* by Shah et al. described a deep learning algorithm capable of identifying loss-of-pulse states using smartwatch signals, with a sensitivity of 67.2% and a low false-alarm rate under simulated conditions. These findings represent an early proof-of-concept for direct detection of out-of-hospital cardiac arrest (58).

Beyond arrhythmia detection, the most translationally realistic near-term role for consumer-grade AI in SCD prevention is continuous, passive tracking of the modifiable lifestyle exposures reviewed in Sections 4–6 — sleep-stage estimation, physical-activity intensity zones, resting heart-rate trends, and adherence to behavioral prescriptions — rather than direct arrhythmic-event prediction, for which discrimination in unselected populations remains modest. Framed this way, wearable-derived signals become inputs to behavior-change support and clinician review rather than autonomous prediction engines. This narrower framing avoids the over-generalization critiqued in recent commentary and aligns with regulator-endorsed use cases. (Evidence tier: 3 for arrhythmic detection performance; 4 for behavior-change intermediate endpoints.).

Despite these advances, important challenges remain. Many AI-based models lack prospective external validation, and performance variability across populations has been reported. A 2025 study by Fiorina et al. (European Heart Journal) demonstrated high predictive accuracy for short-term ventricular arrhythmia risk (AUROC ∼0.95) in 247,254 ambulatory ECG recordings, including external validation (59). In addition, multimodal AI approaches integrating ECG, imaging, and clinical data — such as those reported by Lai et al. in hypertrophic cardiomyopathy — may further enhance predictive performance (60).

Overall, wearable-derived monitoring of lifestyle exposures and AI-augmented ECG risk estimation are complementary lines of work that may inform future SCD prevention research, contingent on prospective external validation and implementation studies.

### 7.5 Public health and systems-level implications

Translating individual-level lifestyle interventions into population-level reductions in SCD requires coordinated, systems-level approaches. The *Lancet* Commission on sudden cardiac death highlights the importance of interdisciplinary collaboration among cardiology, public health, urban planning, and digital health sectors (2).

Lifestyle-based interventions may be particularly relevant in low- and middle-income countries (LMICs), where the burden of SCD is highest and access to device-based prevention strategies such as ICDs is limited (3, 7). Interventions targeting sleep health, OSA screening, physical activity promotion, tobacco control, dietary improvement, and mental health support may offer scalable and cost-effective strategies for risk reduction.

From a health economic perspective, primary prevention ICD therapy has been estimated to cost approximately $34,000–$70,000 per quality-adjusted life year (QALY) gained (61). In contrast, lifestyle interventions are generally considered more cost-effective, although direct comparative analyses remain limited.

Health system structure may influence implementation feasibility. Integrated systems — such as the UK National Health Service, Nordic single-payer models, and national insurance systems in Korea and Taiwan — may be better positioned to incorporate lifestyle-based risk assessment into routine care. In more fragmented systems, implementation may require enhanced coordination across care settings.

Pragmatic implementation research is therefore needed to translate existing evidence into measurable reductions in SCD at the population level.

## 8 Limitations and future research directions

This narrative review is subject to several important limitations. First, as with all narrative reviews, our synthesis is subject to selection bias; inclusion was not governed by a pre-registered protocol or PRISMA-compliant methodology. Future evidence synthesis should consider formal systematic review and network meta-analysis approaches.

Second, the observational nature of the majority of studies reviewed precludes definitive causal inference. Residual confounding by socioeconomic status, comorbidity burden, medication use, and unmeasured lifestyle covariates remains a substantive concern. Mendelian randomization studies have provided causal support for several associations reviewed but are not yet available across all lifestyle-SCD relationships (22).

Third, randomized controlled trials with SCD as a primary endpoint are largely absent from the lifestyle intervention literature. The low incidence of SCD necessitates prohibitively large sample sizes or prolonged follow-up. Composite cardiovascular endpoints (MACE) are commonly used as surrogates, but extrapolation to SCD reduction is imperfect.

Fourth, the OSA-SCD meta-analysis evidence base is characterized by substantial heterogeneity (I^2^ = 79.3%) and an overall non-significant pooled OR (14). This important methodological nuance should caution against overclaiming certainty about the OSA-SCD relationship, even while the untreated subgroup data (OR 3.87) and the mechanistic evidence remain compelling.

Fifth, sex differences in lifestyle–SCD relationships are inadequately characterized in the existing literature. Women experience approximately half the absolute SCD burden of men but differ mechanistically, with greater representation of non-ischemic substrates, Takotsubo cardiomyopathy (50), and microvascular dysfunction. Obstructive sleep apnea is systematically under-diagnosed in women due to atypical symptom presentation, and exercise-associated SCD events are substantially less common in women than in age-matched men (41). The majority of cohort and registry studies cited in this review enrolled predominantly male populations — particularly within the cardiac arrest, electrophysiology, and sport-related SCD literature — limiting the generalizability of effect estimates to women and obscuring sex-specific intervention targets.

Sixth, the generalizability of existing evidence across ethnic and age groups deserves additional scrutiny. The majority of large cohort studies were conducted in predominantly European or North American populations. AI-ECG models trained on Western populations may exhibit attenuated performance in Asian and African populations — a critical limitation given the highest absolute SCD burdens are concentrated in Asia and sub-Saharan Africa (21). Future research priorities include: randomized trials with hard SCD endpoints; Mendelian randomization studies to confirm causal relationships; validation of AI-ECG models across diverse ethnic populations; sex-stratified analyses with adequate representation of women in lifestyle-SCD intervention trials; and equitable access to wearable monitoring technologies in low- and middle-income countries.

This synthesis has deliberate limits. Direct-SCD (Tier 1) evidence is sparse for most lifestyle exposures; much of the quantitative signal reviewed here rests on cardiovascular-mortality proxies (Tier 2) or arrhythmic surrogates (Tier 3). Reverse causality, residual confounding by socioeconomic and psychiatric comorbidity, and imprecise SCD ascertainment are recurring threats to interpretation. We accordingly frame the integrated lifestyle–arrhythmogenic pathway framework as a conceptual scaffold to guide future prospective work, not as a settled causal model on which practice-changing recommendations can already be built.

## 9 Conclusion

Sudden cardiac death is neither inevitable nor wholly unpredictable. The evidence assembled in this review indicates that sleep health, physical activity, diet, psychological well-being, and the avoidance of tobacco and excessive alcohol consumption each contribute — independently and additively — to reducing the risk of lethal ventricular arrhythmia. Sleep disturbance, and untreated OSA in particular, stands out as a substantially underrecognized risk factor: the magnitude of risk in untreated patients, paired with the mechanistic plausibility of intermittent hypoxia and sympathetic dysregulation, makes a strong case for routine screening and timely treatment. Physical activity exhibits an actionable U-shaped relationship with SCD risk, and recent accelerometer-based evidence shows that even brief bouts of vigorous incidental activity confer measurable cardioprotection.

From a clinical standpoint, integrating sleep screening, pre-exercise cardiac evaluation, Mediterranean dietary counseling, psychological-stress management, and smoking-cessation support into routine cardiovascular risk assessment offers a low-cost, high-impact prevention strategy. Emerging evidence supporting AI-enabled ECG analysis and consumer wearable devices for SCD-risk stratification and continuous monitoring of lifestyle-driven risk factors opens a credible path toward precision prevention at scale.

On the basis of the evidence reviewed here, we propose five practical recommendations for clinical implementation. First, sleep history and validated screening for obstructive sleep apnea (e.g., the STOP-BANG questionnaire) should be incorporated into routine cardiovascular risk consultations, with polysomnography offered to high-risk individuals and CPAP therapy initiated where moderate-to-severe OSA is confirmed. Second, patients should be counseled to integrate brief vigorous incidental physical activity (approximately four minutes per day) into daily routines as a low-barrier complement to — or substitute for — structured exercise programs. Third, Mediterranean-style dietary counseling should be initiated at the first cardiovascular risk consultation rather than reserved for secondary prevention. Fourth, screening for chronic psychological stress, depression, and social isolation should be integrated into standard cardiovascular care, with onward referral for cognitive-behavioral intervention or social-prescribing pathways where appropriate. Fifth, formal smoking cessation support — combining first-line pharmacotherapy with behavioral counseling — should be offered to all current smokers with cardiovascular risk factors. These recommendations are pragmatically implementable in most healthcare settings and individually supported by evidence reviewed in the preceding sections.

Future research priorities include: randomized trials powered to detect lifestyle intervention effects on SCD endpoints; Mendelian randomization studies to strengthen causal inference; cross-ethnic validation of AI-ECG risk prediction models; and equitable global access to digital health monitoring technologies.

## References

[1] World Health Organization. WHO Scientific group on sudden cardiac death. World Health Organ Tech Rep Ser. (1985) 726:5–25. 3936284

[2] Marijon E Narayanan K Smith K Barra S Basso C Blom MT. The lancet commission to reduce the global burden of sudden cardiac death: a call for multidisciplinary action. Lancet. (2023) 402:883–936. 10.1016/S0140-6736(23)00875-9 37647926

[3] Hua W Zhang L-F Wu Y-F Liu X-Q Guo D-S Zhou H-L. Incidence of sudden cardiac death in China: analysis of 4 regional populations. J Am Coll Cardiol. (2009) 54:1110–8. 10.1016/j.jacc.2009.06.016 19744622

[4] Zeppenfeld K Tfelt-Hansen J De Riva M Winkel BG Behr ER Blom NA. 2022 ESC guidelines for the management of patients with ventricular arrhythmias and the prevention of sudden cardiac death. Eur Heart J. (2022) 43:3997–4126. 10.1093/eurheartj/ehac262 36017572

[5] Skjelbred T Warming PE Krøll J Andersen MP Torp-Pedersen C Winkel BG. Sudden cardiac death as first manifestation of cardiovascular disease: a nationwide study of 54,028 deaths. JACC Clin Electrophysiol. (2025) 11:881–90. 10.1016/j.jacep.2024.12.012 39918459

[6] Bagnall RD Weintraub RG Ingles J Duflou J Yeates L Lam L. A prospective study of sudden cardiac death among children and young adults. N Engl J Med. (2016) 374:2441–52. 10.1056/NEJMoa1510687 27332903

[7] Magnussen C Ojeda FM Leong DP Alegre-Diaz J Amouyel P Aviles-Santa L. Global effect of modifiable risk factors on cardiovascular disease and mortality. N Engl J Med. (2023) 389:1273–85. 10.1056/NEJMoa2206916 37632466 PMC10589462

[8] Lloyd-Jones DM Allen NB Anderson CAM Black T Brewer LPC Foraker RE. Life’s essential 8: updating and enhancing the American Heart Association’s construct of cardiovascular health: a presidential advisory from the American Heart Association. Circulation. (2022) 146:e18–43. 10.1161/CIR.0000000000001078 35766027 PMC10503546

[9] Gami AS Howard DE Olson EJ Somers VK. Day–night pattern of sudden death in obstructive sleep apnea. N Engl J Med. (2005) 352:1206–14. 10.1056/NEJMoa041832 15788497

[10] Willich SN Maclure M Mittleman M Arntz HR Muller JE. Sudden cardiac death: support for a role of triggering in causation. Circulation. (1993) 87:1442–50. 10.1161/01.CIR.87.5.1442 8490998

[11] Grandner MA. Sleep, health, and society. Sleep Med Clin. (2017) 12:1–22. 10.1016/j.jsmc.2016.10.012 28159089 PMC6203594

[12] Lavie CJ Arena R Swift DL Johannsen NM Sui X Lee DC. Exercise and the cardiovascular system: clinical science and cardiovascular outcomes. Circ Res. (2015) 117:207–19. 10.1161/CIRCRESAHA.117.305205 26139859 PMC4493772

[13] Estruch R Ros E Salas-Salvadó J Covas M-I Corella D Arós F. Primary prevention of cardiovascular disease with a Mediterranean diet supplemented with extra-virgin olive oil or nuts. N Engl J Med. (2018) 378:e34. 10.1056/NEJMoa1800389 29897866

[14] Zou X Zhou X Yi S. Obstructive sleep apnea and the risk of sudden cardiac death: a systematic review and meta-analysis. BMC Cardiovasc Disord. (2025) 25:751. 10.1186/s12872-025-05193-7 41120867 PMC12542175

[15] La Rovere MT Bigger JT Marcus FI Mortara A Schwartz PJ, ATRAMI (Autonomic Tone and Reflexes After Myocardial Infarction) Investigators. Baroreflex sensitivity and heart-rate variability in prediction of total cardiac mortality after myocardial infarction. Lancet. (1998) 351:478–84. 10.1016/s0140-6736(97)11144-8 9482439

[16] Finocchiaro G Westaby J Sheppard MN Papadakis M Sharma S. Sudden cardiac death in young athletes. JACC State-of-the-Art Review. J Am Coll Cardiol. (2024) 83:350–70. 10.1016/j.jacc.2023.10.032 38199713

[17] Steptoe A Kivimäki M. Stress and cardiovascular disease. Nat Rev Cardiol. (2012) 9:360–70. 10.1038/nrcardio.2012.45 22473079

[18] Ridker PM Everett BM Thuren T MacFadyen JG Chang WH Ballantyne C. Antiinflammatory therapy with canakinumab for atherosclerotic disease. N Engl J Med. (2017) 377:1119–31. 10.1056/NEJMoa1707914 28845751

[19] Mewton N Liu CY Croisille P Bluemke D Lima JAC. Assessment of myocardial fibrosis with cardiovascular magnetic resonance. J Am Coll Cardiol. (2011) 57:891–903. 10.1016/j.jacc.2010.11.013 21329834 PMC3081658

[20] La Gerche A Burns AT Mooney DJ Inder WJ Taylor AJ Bogaert J. Exercise-induced right ventricular dysfunction and structural remodeling in endurance athletes. Eur Heart J. (2012) 33:998–1006. 10.1093/eurheartj/ehr397 22160404

[21] Kolk MZH Ruipérez-Campillo S Wilde AAM Knops RE Narayan SM Tjong FVY. Prediction of sudden cardiac death using artificial intelligence: current status and future directions. Heart Rhythm. (2025) 22:756–66. 10.1016/j.hrthm.2024.09.003 39245250 PMC12057726

[22] Wang S Li Z Wang X Guo S Sun Y Li G. Associations between sleep duration and cardiovascular diseases: a meta-review and meta-analysis of observational and Mendelian randomization studies. Front Cardiovasc Med. (2022) 9:930000. 10.3389/fcvm.2022.930000 36035915 PMC9403140

[23] Reutrakul S Van Cauter E. Sleep influences on obesity, insulin resistance, and risk of type 2 diabetes. Metab Clin Exp. (2018) 84:56–66. 10.1016/j.metabol.2018.02.010 29510179

[24] Nambiema A Lisan Q Vaucher J Perier M-C Boutouyrie P Danchin N. Healthy sleep score changes and incident cardiovascular disease in European prospective community-based cohorts. Eur Heart J. (2023) 44:4968–78. 10.1093/eurheartj/ehad657 37860848 PMC10719494

[25] Kwok CS Kontopantelis E Kuligowski G Gray M Muhyaldeen A Gale CP. Self-reported sleep duration and quality and cardiovascular disease and mortality: a dose-response meta-analysis. J Am Heart Assoc. (2018) 7:e008552. 10.1161/JAHA.118.008552 30371228 PMC6201443

[26] Cappuccio FP Cooper D D'Elia L Strazzullo P Miller MA. Sleep duration predicts cardiovascular outcomes: a systematic review and meta-analysis of prospective studies. Eur Heart J. (2011) 32:1484–92. 10.1093/eurheartj/ehr007 21300732

[27] Huang Y-M Xia W Ge Y-J Hou J-H Tan L Xu W. Sleep duration and risk of cardio-cerebrovascular disease: a dose-response meta-analysis of cohort studies comprising 3.8 million participants. Front Cardiovasc Med. (2022) 9:907990. 10.3389/fcvm.2022.907990 36237900 PMC9551171

[28] Arora N Brumpton BM Åsvold BO Loennechen JP Malmo V Bhatta L. A Mendelian randomization study investigating the role of sleep traits and their joint effects on the incidence of atrial fibrillation. Eur J Prev Cardiol. (2025) zwaf062. 10.1093/eurjpc/zwaf062 39913680

[29] Besedovsky L Lange T Haack M. The sleep–immune crosstalk in health and disease. Physiol Rev. (2019) 99:1325–80. 10.1152/physrev.00010.2018 30920354 PMC6689741

[30] Muller JE Stone PH Turi ZG Rutherford JD Czeisler CA Parker C. Circadian variation in the frequency of onset of acute myocardial infarction. N Engl J Med. (1985) 313:1315–22. 10.1056/NEJM198511213132103 2865677

[31] Gami AS Olson EJ Shen WK Wright RS Ballman KV Hodge DO. Obstructive sleep apnea and the risk of sudden cardiac death: a longitudinal study of 10,701 adults. J Am Coll Cardiol. (2013) 62:610–6. 10.1016/j.jacc.2013.04.080 23770166 PMC3851022

[32] Ryan CM Usui K Floras JS Bradley TD. Effect of continuous positive airway pressure on ventricular ectopy in heart failure patients with obstructive sleep apnoea. Thorax. (2005) 60:781–5. 10.1136/thx.2005.040972 15994252 PMC1747520

[33] Harbison J O'Reilly P McNicholas WT. Cardiac rhythm disturbances in the obstructive sleep apnea syndrome: effects of nasal continuous positive airway pressure therapy. Chest. (2000) 118:591–5. 10.1378/chest.118.3.591 10988177

[34] Thompson PD. Exercise and physical activity in the prevention and treatment of atherosclerotic cardiovascular disease. Arterioscler Thromb Vasc Biol. (2003) 23:1319–21. 10.1161/01.ATV.0000087143.33998.F2 12909570

[35] Wahid A Manek N Nichols M Kelly P Foster C Webster P. Quantifying the association between physical activity and cardiovascular disease and diabetes: a systematic review and meta-analysis. J Am Heart Assoc. (2016) 5:e002495. 10.1161/JAHA.115.002495 27628572 PMC5079002

[36] Biswas A Oh PI Faulkner GE Bajaj RR Silver MA Mitchell MS. Sedentary time and its association with risk for disease incidence, mortality, and hospitalization in adults: a systematic review and meta-analysis. Ann Intern Med. (2015) 162:123–32. 10.7326/M14-1651 25599350

[37] Kim JH Rim AJ Miller JT Jackson M Patel N Rajesh S. Cardiac arrest during long-distance running races. JAMA. (2025) 333:1699–707. 10.1001/jama.2025.3026 40159341 PMC11955904

[38] Javed W Botis I Goh ZM Shabi M Brown B Tomoaia R. Ventricular arrhythmia and cardiac fibrosis in endurance experienced athletes (VENTOUX). Circ Cardiovasc Imaging. (2025) 18:e018470. 10.1161/CIRCIMAGING.125.018470 40675176 PMC12356567

[39] Stamatakis E Ahmadi MN Gill JMR Thøgersen-Ntoumani C Gibala MJ Doherty A. Association of wearable device-measured vigorous intermittent lifestyle physical activity with mortality. Nat Med. (2022) 28:2521–9. 10.1038/s41591-022-02100-x 36482104 PMC9800274

[40] Strain T Wijndaele K Dempsey PC Sharp SJ Pearce M Jeon J. Wearable-device-measured physical activity and future health risk. Nat Med. (2020) 26:1385–91. 10.1038/s41591-020-1012-3 32807930 PMC7116559

[41] Thompson PD Franklin BA Balady GJ Blair SN Corrado D Estes NAM. Exercise and acute cardiovascular events: placing the risks into perspective. A scientific statement from the American Heart Association council on nutrition, physical activity, and metabolism and the council on clinical cardiology. Circulation. (2007) 115:2358–68. 10.1161/CIRCULATIONAHA.107.181485 17468391

[42] Lampert R Ackerman MJ Marino BS Burg M Ainsworth B Salberg L. Vigorous exercise in patients with hypertrophic cardiomyopathy. JAMA Cardiol. (2023) 8:595–605. 10.1001/jamacardio.2023.1042 37195701 PMC10193262

[43] Prasad DS Kabir Z Dash AK Das BC. Smoking and cardiovascular health: a review of the epidemiology, pathogenesis, prevention and control of tobacco. Indian J Med Sci. (2009) 63:520–33. 10.4103/0019-5359.58884 20075556

[44] D'Alessandro A Boeckelmann I Hammwöhner M Goette A. Nicotine, cigarette smoking and cardiac arrhythmia: an overview. Eur J Prev Cardiol. (2012) 19:297–305. 10.1177/1741826711411738 22779085

[45] Ettinger PO Wu CF De La Cruz C Weisse AB Ahmed SS Regan TJ. Arrhythmias and the “holiday heart": alcohol-associated cardiac rhythm disorders. Am Heart J. (1978) 95:555–62. 10.1016/0002-8703(78)90296-x 636996

[46] Piano MR. Alcoholic cardiomyopathy: incidence, clinical characteristics, and pathophysiology. Chest. (2002) 121:1638–50. 10.1378/chest.121.5.1638 12006456

[47] Griswold MG Fullman N Hawley C Arian N Zimsen SRM Tymeson HD. Alcohol use and burden for 195 countries and territories, 1990–2016: a systematic analysis for the global burden of disease study 2016. Lancet. (2018) 392:1015–35. 10.1016/S0140-6736(18)31310-2 30146330 PMC6148333

[48] Anderson BO Berdzuli N Ilbawi A Kestel D Kluge HP Krech R. Health and cancer risks associated with low levels of alcohol consumption. Lancet Public Health. (2023) 8:e6–7. 10.1016/S2468-2667(22)00317-6 36603913 PMC9831798

[49] Zhao J Stockwell T Naimi T Churchill S Clay J Sherk A. Association between daily alcohol intake and risk of all-cause mortality: a systematic review and meta-analyses. JAMA Netw Open. (2023) 6:e236185. 10.1001/jamanetworkopen.2023.6185 37000449 PMC10066463

[50] Templin C Ghadri JR Diekmann J Napp LC Bataiosu DR Jaguszewski M. Clinical features and outcomes of takotsubo (stress) cardiomyopathy. N Engl J Med. (2015) 373:929–38. 10.1056/NEJMoa1406761 26332547

[51] Steinberg JS Arshad A Kowalski M Kukar A Suma V Vloka M. Increased incidence of life-threatening ventricular arrhythmias in implantable defibrillator patients after the world trade center attack. J Am Coll Cardiol. (2004) 44:1261–4. 10.1016/j.jacc.2004.06.032 15364329

[52] Mozaffarian D Psaty BM Rimm EB Lemaitre RN Burke GL Lyles MF. Fish intake and risk of incident atrial fibrillation. Circulation. (2004) 110:368–73. 10.1161/01.CIR.0000138154.00779.A5 15262826 PMC1201400

[53] Yeghiazarians Y Jneid H Tietjens JR Redline S Brown DL El-Sherif N. Obstructive sleep apnea and cardiovascular disease: a scientific statement from the American Heart Association. Circulation. (2021) 144:e56–67. 10.1161/CIR.0000000000000988 34148375

[54] Mehra R Chung MK Olshansky B Dobrev D Jackson CL Kundel V. Sleep-disordered breathing and cardiac arrhythmias in adults: mechanistic insights and clinical implications: a scientific statement from the American Heart Association. Circulation. (2022) 146:e119–36. 10.1161/CIR.0000000000001082 35912643 PMC10227720

[55] Gottlieb DJ Punjabi NM. Diagnosis and management of obstructive sleep apnea: a review. JAMA. (2020) 323:1389–400. 10.1001/jama.2020.3514 32286648

[56] Perez MV Mahaffey KW Hedlin H Rumsfeld JS Garcia A Ferris T. Large-scale assessment of a smartwatch to identify atrial fibrillation. N Engl J Med. (2019) 381:1909–17. 10.1056/NEJMoa1901183 31722151 PMC8112605

[57] van Steijn NJ Blommestijn IS Blok S Pepplinkhuizen S Somsen GA Knops RE. Enhanced detection and prompt diagnosis of atrial fibrillation using apple watch: a randomized controlled trial. J Am Coll Cardiol. (2026) 87:1714–28. 10.1016/j.jacc.2025.11.032 41569211

[58] Shah K Wang A Chen Y Munjal J Chhabra S Stange A. Automated loss of pulse detection on a consumer smartwatch. Nature. (2025) 642:174–81. 10.1038/s41586-025-08810-9 40010378 PMC12137114

[59] Fiorina L Carbonati T Narayanan K Li J Henry C Singh JP. Near-term prediction of sustained ventricular arrhythmias applying artificial intelligence to single-lead ambulatory electrocardiogram. Eur Heart J. (2025) 46:1998–2008. 10.1093/eurheartj/ehaf073 40157386 PMC12127732

[60] Lai C Yin M Kholmovski EG Popescu DM Lu D-Y Scherer E. Multimodal artificial intelligence to forecast arrhythmic death in hypertrophic cardiomyopathy. Nat Cardiovasc Res. (2025) 4:891–903. 10.1038/s44161-025-00679-1 40603582 PMC12259465

[61] Sanders GD Hlatky MA Owens DK. Cost-effectiveness of implantable cardioverter–defibrillators. N Engl J Med. (2005) 353:1471–80. 10.1056/NEJMsa051989 16207849

[62] Truyen TTT Lin H Mathias M Chugh H Reinier K Benjamin EJ. Validation of a novel risk prediction score for sudden cardiac death in the framingham heart study. Circ Arrhythm Electrophysiol. (2025) 18(6):e013647. 10.1161/CIRCEP.124.013647 40391444 PMC12173765

[63] Truyen TTT Pham VNA Nguyen HDT. Beyond cardiac risk factors: non-cardiovascular comorbidities in sudden cardiac death prediction. Front Cardiovasc Med. (2026) 13:1728987. 10.3389/fcvm.2026.1728987 41657481 PMC12872818

[64] La Gerche A Wasfy MM Brosnan MJ Claessen G Fatkin D Heidbuchel H. The athlete’s heart—challenges and controversies: jACC focus seminar 4/4. J Am Coll Cardiol. (2022) 80(14):1346–62. 10.1016/j.jacc.2022.07.014 36075838

[65] Vo J Truyen TTT Uy-Evanado A Sargsyan A Chugh H Young C. Sudden cardiac death associated with fatty liver disease. Int J Cardiol Heart Vasc. (2025) 56:101602. 10.1016/j.ijcha.2025.101602 39867850 PMC11759637

