# Supplementary material for: Modifiable lifestyle factors and sudden cardiac death: mechanisms, evidence, and prevention strategies
Source: Front Cardiovasc Med. 2026 Jul 29;13:1870365. doi: 10.3389/fcvm.2026.1870365 (PMC13461472; doi:10.3389/fcvm.2026.1870365)
Supplement: Supplementary file 1 [file Datasheet1.pdf]

Supplementary Material S1

Detailed Literature Search Strategy and Study Selection Flow

Companion to: "Modifiable Lifestyle Factors and Sudden Cardiac Death: Mechanisms, Evidence, and Prevention Strategies"

S1.1 Database-Specific Search Strings

A two-stage literature search was conducted. The original search was executed on 22 March 2025 across PubMed/MEDLINE, Web of Science Core Collection, and EMBASE, covering English-language publications dated 1 January 2003 – 31 March 2025 (PubMed/MEDLINE and Web of Science Core Collection yielded 7,455 and 5,158 records, respectively, on re-execution of the cited Boolean strings within the specified date window; for EMBASE, per-database counts were not separately recorded, as the analysis was conducted on the deduplicated combined dataset — see screening flow in S1.3). To ensure currency at the time of submission, a supplementary verification search was conducted on 28 April 2026, using the same Boolean strategy in PubMed/MEDLINE restricted to publications dated 1 April 2025 – 28 April 2026; this returned 316 records. After title/abstract screening of the verification yield, seven high-impact publications were retained and incorporated into the manuscript: Arora et al. (Eur J Prev Cardiol, 2025 — primary Mendelian randomization of sleep traits and atrial fibrillation), Kim et al. (JAMA, 2025 — cardiac arrest in long-distance running races, n = 29.3 million finishers), Javed et al. (Circ Cardiovasc Imaging, 2025 — VENTOUX cohort of endurance athletes with cardiac fibrosis and ventricular arrhythmia), Fiorina et al. (Eur Heart J, 2025 — large-scale external validation of an AI-ECG model for near-term ventricular arrhythmia prediction in 247,254 ambulatory ECG recordings), van Steijn et al. (J Am Coll Cardiol, 2026 — the EQUAL randomized controlled trial of Apple Watch–based atrial fibrillation screening in older adults), Shah et al. (Nature, 2025 — automated loss-of-pulse detection on a consumer smartwatch), and Lai et al. (Nat Cardiovasc Res, 2025 — multimodal AI for arrhythmic-death forecasting in hypertrophic cardiomyopathy). The two-stage approach is reported transparently here so that the timeline between original search execution, manuscript preparation, and submission can be evaluated by reviewers.

PubMed/MEDLINE (search executed: 22 March 2025)

```
("sudden cardiac death"[MeSH] OR "sudden cardiac death"[tiab] OR "ventricular fibrillation"[MeSH] OR "ventricular fibrillation"[tiab] OR "ventricular tachyarrhythmia"[tiab] OR "out-of-hospital cardiac arrest"[tiab]) AND ("sleep duration"[tiab] OR "sleep apnoea"[tiab] OR "sleep apnea"[tiab] OR "obstructive sleep apnoea"[tiab] OR "OSA"[tiab] OR "circadian rhythm"[MeSH] OR "shift work"[tiab] OR "physical activity"[MeSH] OR "exercise"[MeSH] OR "vigorous physical activity"[tiab] OR "incidental physical activity"[tiab] OR "Mediterranean diet"[MeSH] OR "dietary pattern"[tiab] OR "psychological stress"[tiab] OR "depression"[MeSH] OR "smoking"[MeSH] OR "tobacco"[MeSH] OR "alcohol drinking"[MeSH] OR "sedentary behavior"[MeSH] OR "wearable electronic devices"[MeSH] OR "artificial intelligence"[MeSH] OR "electrocardiography"[MeSH]) AND ("2003/01/01"[PDAT] : "2025/03/31"[PDAT]) AND English[lang]
```

Web of Science Core Collection (search executed: 22 March 2025)

```
TS=("sudden cardiac death" OR "ventricular fibrillation" OR "ventricular tachyarrhythmia" OR "out-of-hospital cardiac arrest") AND TS=("sleep duration" OR "sleep apnoea" OR "sleep apnea" OR "obstructive sleep apnoea" OR "OSA" OR "circadian rhythm" OR "shift work" OR "physical activity" OR "exercise" OR "vigorous physical activity" OR "incidental physical activity" OR "Mediterranean diet" OR "dietary pattern" OR "psychological stress" OR "depression" OR "smoking" OR "tobacco" OR "alcohol" OR "sedentary behaviour" OR "wearable" OR "artificial intelligence" OR "electrocardiography") AND PY=2003-2025 AND DT=(Article OR Review) AND LA=English
```

EMBASE (search executed: 22 March 2025, Ovid syntax)

```
("sudden cardiac death"/exp OR "sudden cardiac death":ti,ab OR "ventricular fibrillation"/exp OR "ventricular tachyarrhythmia":ti,ab OR "out of hospital cardiac arrest":ti,ab) AND ("sleep duration":ti,ab OR "obstructive sleep apnea"/exp OR "obstructive sleep apnoea":ti,ab OR "circadian rhythm"/exp OR "shift work":ti,ab OR "physical activity"/exp OR exercise/exp OR "vigorous physical activity":ti,ab OR "incidental physical activity":ti,ab OR "Mediterranean diet"/exp OR "dietary pattern":ti,ab OR "psychological stress"/exp OR depression/exp OR smoking/exp OR tobacco/exp OR "alcohol consumption"/exp OR "sedentary behavior"/exp OR "wearable device"/exp OR "artificial intelligence"/exp OR electrocardiography/exp) AND [2003-2025]/py AND [english]/lim AND ([article]/lim OR [review]/lim)
```

S1.2 Inclusion and Exclusion Criteria

Inclusion criteria (one or more):

- Prospective or retrospective cohort study with cardiovascular mortality, sudden cardiac death, ventricular arrhythmia, atrial fibrillation, or related cardiac endpoints.
- Randomized controlled trial (RCT) of a lifestyle, behavioral, pharmacological, or device intervention with cardiovascular outcomes.
- Systematic review or meta-analysis on lifestyle exposures and cardiovascular endpoints.
- Mendelian randomization analysis of a lifestyle exposure and a cardiovascular endpoint.
- Clinical practice guideline or scientific statement from ESC, AHA, ACC, or WHO.
- Mechanistic study (animal, ex vivo, or clinical physiology) directly relevant to one of the four arrhythmogenic pathways discussed in the review.
- Foundational landmark publications (specifically retained outside the 2003–2025 search window where they remain primary sources for established concepts; see S1.5).

Exclusion criteria:

- Case reports, conference abstracts only, letters to the editor without primary data, editorials, and commentary pieces.
- Studies in non-human models without explicit translational discussion.
- Studies focused on secondary prevention in established cardiac arrest survivors only (without a primary prevention component).
- Non-English language publications (acknowledged limitation; see Section 8 of the main manuscript).
- Duplicate publications and overlapping cohort analyses where the most recent or most complete version was already included.

S1.3 Study Selection Overview (Narrative Framework)

This is a narrative review and full PRISMA compliance is neither claimed nor required. To improve transparency, we provide an approximate overview of the screening process below; the figures should be read as order-of-magnitude indicators of the breadth of the search rather than precise audit-trail counts. Title and abstract screening was performed by the lead author (JZ) with disagreements resolved through discussion with a second reviewer (HS). Final full-text inclusion decisions were made jointly by all authors.

Stage	Records
Records identified through PubMed/MEDLINE (verified yield, Boolean string in S1.1)	7,455
Records identified through Web of Science Core Collection (verified yield)	5,158
Records identified through EMBASE (search executed; per-database count not separately recorded — see footnote)	—†
Records identified through hand-searching of cited references (approximate)	~30†
Foundational publications retained outside search window (see S1.5)	8

Combined records identified across the three databases (PubMed and WoS verified; EMBASE included in subsequent deduplication)	~13,000+†
Records remaining after duplicate removal of the combined dataset (approximate)	~9,500†
Records advancing past title/abstract screening (approximate)	~250†
Records reviewed at full-text level (approximate)	~80†
Common reasons for full-text exclusion	case reports / commentary / editorials; secondary-prevention-only studies; non-cardiovascular endpoints; late-identified non-English; duplicate or overlapping cohorts
Studies retained for narrative synthesis	~80
References retained after first-round screening (initial March 2025 search)	51
References added during internal peer-review revision (alcohol section)	3
References added from April 2026 verification search (high-impact 2025–2026 papers)	7
Reviewer-requested references (Reviewer 4)	4
Final reference count (total, after Vancouver renumbering)	65

† PubMed and Web of Science yields were verified by re-execution of the search strategy reported in S1.1 within the date window 1 January 2003 – 31 March 2025. For EMBASE, per-database counts were not separately recorded, as the analysis was conducted on the deduplicated combined dataset. Downstream figures (records after deduplication, after title/abstract screening, and reviewed at full-text level) are reported as order-of-magnitude indicators consistent with narrative-review practice rather than precise audit-trail counts.

Of the 51 records originally retained through the March 2025 database searches, three additional references on contemporary alcohol-cardiovascular evidence were added during internal peer-review revision (corresponding to the GBD 2016 Alcohol Collaborators report, Lancet 2018; Zhao et al., JAMA Network Open 2023; and Anderson et al., Lancet Public Health 2023), and seven further high-impact references were incorporated from the April 2026 verification search (full citations in S1.1),together with four additional reviewer-requested references (the sudden-cardiac-death risk-score validation, non-cardiovascular comorbidities, athlete's heart, and steatotic-liver-disease references), added at the request of Reviewer 4. After Vancouver-order renumbering, the final reference count is 65.

S1.4 PRISMA-S Search-Reporting Checklist (abbreviated)

Item	Status / Location
Database name(s)	PubMed/MEDLINE, Web of Science Core Collection, EMBASE — reported in main text §2 and S1.1
Search interface (platform)	PubMed via NLM; Web of Science via Clarivate; EMBASE via Ovid — reported in S1.1
Date of last search	22 March 2025 — reported in S1.1
Date range searched	1 January 2003 – 31 March 2025 — reported in main text §2
Full Boolean strings	Reported in S1.1 (this document)
Inclusion / exclusion criteria	Reported in S1.2
Selection process (single vs dual reviewer)	Reported in S1.3 (single screener with second-reviewer adjudication for disagreements)
Records flow (retrieved → screened → included)	Reported in S1.3 flow table
Reasons for exclusion at full-text stage	Reported in S1.3
Foundational publications outside search window	Reported in S1.5 with explicit justification
Language restriction	English only — limitation explicitly acknowledged in main text

	§8 and S1.6
Risk-of-bias appraisal	Not systematically applied; consistent with narrative review methodology — acknowledged in S1.6

S1.5 Foundational Publications Retained Outside the Search Window

The formal search window of 1 January 2003 – 31 March 2025 was applied to ensure currency of the included evidence base. However, the following foundational publications, predating 2003, were specifically retained because they remain the primary cited source for established clinical or epidemiological concepts that have not been superseded by post-2003 work. Their inclusion is a deliberate methodological choice reflecting the requirements of a narrative review of long-standing clinical phenomena.

Reference (manuscript number)	Year	Justification for retention
Ettinger PO et al., Holiday-heart syndrome, Am Heart J (Ref 45)	1978	Original primary description of the clinical syndrome; remains the most cited source.
WHO Scientific Group on Sudden Cardiac Death, WHO Tech Rep Ser (Ref 1)	1985	Foundational WHO definition of SCD; superseded operationally by ESC 2022 (Ref 4) but retained as historical reference.
Muller JE et al., Circadian variation of acute MI, NEJM (Ref 30)	1985	Original demonstration of morning peak in cardiovascular events; primary source for circadian-arrhythmia link.
Willich SN et al., SCD and triggering, Circulation (Ref 10)	1993	Foundational evidence that SCD is precipitated by acute triggers; primary source for the trigger framework.
La Rovere MT et al., ATRAMI, Lancet (Ref 15)	1998	Landmark prospective cohort establishing baroreflex sensitivity and HRV as autonomic predictors of cardiac mortality post-MI.
Harbison J et al., OSA and cardiac rhythm, Chest (Ref 33)	2000	Primary demonstration that nasal CPAP reduces nocturnal arrhythmia in OSA; foundational therapeutic-mechanism study.
Piano MR, Alcoholic cardiomyopathy, Chest (Ref 46)	2002	Definitive review of alcoholic cardiomyopathy phenotype and pathophysiology; primary clinical reference for the entity.
Thompson PD, Exercise and atherosclerosis, ATVB (Ref 34)	2003	On the cusp of the search window (January 2003); foundational AHA-aligned review of exercise-cardiovascular relationships.

These studies are cited for conceptual and historical completeness rather than for quantitative effect estimation; contemporary effect estimates are drawn from the post-2003 evidence base identified through the formal search.

S1.6 Methodological Limitations of the Search Strategy

The principal limitations of the search strategy, acknowledged here for transparency:

(i) Language bias: Restricting to English-language publications excludes a substantial volume of cardiovascular epidemiology in Chinese, Japanese, Korean, Russian, Arabic, and other languages — particularly relevant for a topic where the highest absolute SCD burden is concentrated in Asia.

(ii) Publication bias: Reliance on three indexed databases (PubMed, Web of Science, EMBASE) may underrepresent grey literature, conference proceedings, and non-indexed registries.

(iii) Single-reviewer screening: Title and abstract screening was performed by a single reviewer with second-reviewer adjudication only for disagreements, in line with narrative-review practice but below the dual-reviewer standard for systematic reviews.

(iv) Heterogeneous quality appraisal: Formal risk-of-bias tools (e.g., Cochrane RoB 2, ROBINS-I, AMSTAR-2) were not systematically applied to each included study; quality was instead reflected in the narrative discussion of effect sizes and certainty (consistent with narrative review methodology).

(v) Non-systematic citation tracking: Backward citation searches were performed selectively for

high-yield references rather than systematically, which may have resulted in under-identification of older relevant studies.

These limitations are explicitly discussed in Section 8 of the main manuscript and are intended to scope the inferential weight of the conclusions appropriately.
